# A Concept Analysis of Nurses’ Clinical Decision Making: Implications for Korea

**DOI:** 10.3390/ijerph19063596

**Published:** 2022-03-18

**Authors:** Sunyoung Oh, Minkyung Gu, Sohyune Sok

**Affiliations:** 1 Department of Nursing, Graduate School, Kyung Hee University, Seoul 02447, Korea; powercyte@naver.com; 2 Department of Nursing, College of Science and Technology, Daejin University, Pocheon-si 11159, Korea; g-minkyung@hanmail.net; 3 Department of Nursing, College of Nursing Science, Kyung Hee University, Seoul 02447, Korea

**Keywords:** clinical decision making, concept formation, Korea

## Abstract

The study’s purpose was to identify the meaning and the attributes of Korean nurses’ clinical decision making. A sequential and systematic literature review with reflection according to the conceptual analysis method of Walker and Avant was used in this study. Data sources included the National Assembly Library, the National Digital Science Library, ProQuest, PubMed, MEDLINE, and CINAHL. Finally, twenty-six articles were included in this concept analysis. The concept of Korean nurses’ clinical decision making consisted of the following attributes: clinical reasoning, choosing and applying challenging alternatives, and professional assessment and resetting. Antecedents consisted of: recognizing complex and diverse patient situations with high uncertainty, the need to solve problems according to priority, prior experience in clinical decision making, and interrelationships with fellow medical staff. Consequences consisted of: providing high-quality nursing services, improving the patient’s safety, and increased satisfaction with clinical decision making. Based on these results, the conceptual attributes of Korean nurses’ clinical decision making had slightly different characteristics but were organically interrelated. The results of analyzing the concept of Korean nurses’ clinical decision making provide a better understanding of it and contribute to expanding nursing knowledge and developing a valid and reliable measurement.

## 1. Introduction

In modern society, due to the development of medical technology and the rapid aging of the population, the complexity of nursing tasks in relation to patients with chronic diseases is increasing [1,2]. This requires clinical decision making by skilled and professional nurses [2].

Clinical decision-making nurtures professional nursing knowledge for maintaining the life and promoting the health of a patient [3,4]. Above all, it is essential to solve the priority nursing problem of the patient based on clinical experience [4,5]. The nurse can recognize the patient’s priority health problem based on the patient’s information and can predict the uncertain disease situation of the patient through clinical decision making [2,3]. Therefore, in the clinical practice field, it is necessary for nurses to collect various information needed for the nursing of patients, and to make clinical decisions with rational and critical reasoning [3,6,7].

For nurses, clinical decision-making entails increasing their cognitive abilities and intuition, which will allow them to directly and indirectly identify the problems affecting their patients and select appropriate nursing alternatives for them [3]. Thus, clinical decision making is a key competency required in the field of clinical practice [3]. In addition, it allows the provision of high-quality nursing by consolidating the nursing practices and enabling important judgments to be made in the uncertain disease situation of a patient, thereby contributing to the patient’s comfort [2,8,9].

Nurses in Korea are being educated on following the internationally standardized roles and responsibilities of nurses in the professional role and decision-making process since nursing education first began [4,7]. Nurses are applying them to patient care in actual clinical practice. However, in the decision-making process of Korean nurses, their difference from the nurses of other countries is that their dependence on the decision making of the doctor is excessively high in an independent decision-making situation related to patient care [7]. Moreover, the decision-making results showed that the self-confidence of nurses is low [4]. As a result, independence and autonomy in nursing practice are poor [4,7]. This can be seen as a violation of international standards emphasizing the responsibility and role for patient care as a nursing professional [3,4,7,8]. Passive decision making in Korea is also related to the legal role prescription of nurses stipulated in the current medical law [7,8].

Currently, the most frequently used nursing theory when referring to the clinical decision-making ability of nurses is the clinical decision making four-step model, which summarizes the clinical decision-making process of Tanner [10] in four stages. This model is a formalized theoretical framework constructed from the perspective of instructors who provide nursing education for nursing students. However, the author used this model as a reference material to reconstruct the previous literature with a new concept that can explain the decision-making ability of clinical nurses.

Walker and Avant [11] stated that it is very important to clarify the nature of the field or concept of interest, and to develop it into relevant knowledge. The conceptual analysis method of Walker and Avant [11] has the advantage of clarifying the existing meaning and the possibility to be used for the purpose of developing or adding new definitions.

Thus, it was selected and utilized as it was thought to be suitable for the purpose of this study. Using the conceptual analysis method of Walker and Avant [11] to confirm the nature of the field or concept of interest in this study through multidisciplinary comparison means reinterpreting the clinical decision making of nurses in the current nursing practice field from a new perspective.

In addition, the studies in the domestic and foreign literature that confirm the components of clinical decision making were conducted mainly in the fields of medicine, psychology, business administration, and public administration, and there has been no study that analyzed the concept of clinical decision making in the context of nursing [12,13]. Most previous studies on the clinical decision making of nurses defined correlations based on factors that influence decision making, or classified correlations by including situational diagnostic content [1,3,5]. These previous studies adjudged the clinical decision making of nurses as based on intuition, i.e., on inference or foresight, as well as on recognition of various patient problems in the clinical practice field, and particularly, as covering only causes and causal relationships in clinical situations [4,14,15].

The authors believe that it is necessary to clarify the concept of nurses’ clinical decision making using the conceptual analysis method of Walker and Avant [11]. Moreover, this study will provide important basic data for promoting the development of the nursing practice for the desirable promotion of patient health with the revealed attributes of nurses’ clinical decision making, and above all, for nurturing nurses’ integrated key competencies. The aim of this study was to sequentially and systematically identify the clear attributes of nurses’ clinical decision making according to the conceptual analysis method of Walker and Avant [11]. In addition, model and additional cases were presented, and an attempt was made to derive the antecedents and consequences of nurses’ understanding of clinical decision making.

## 2. Materials and Methods

### 2.1. Study Design

A sequential and systematic literature review with reflection according to the conceptual analysis method of Walker and Avant [11] was used in this study.

### 2.2. Concept Analysis Process

The specific process of the conceptual analysis method of Walker and Avant [11], which, as mentioned above, was used in this study to sequentially and systematically identify the clear attributes of nurses’ clinical decision making in the clinical practice field, is as shown below.

(1) Select the terms related to the concept of interest and confirm the concept’s theoretical definition.
(2) Set the purpose of the concept of interest for data collection.
(3) In relation to the characteristics of the concept of interest, check all the data that can be analyzed to understand its attributes, antecedents, and consequences.
(4) Check the attributes of the concept of interest.
(5) Determine the exact attributes of the concept of interest.
(6) Construct model and additional cases (similar, boundary, and opposite cases), focusing on the identified attributes of the concept of interest.
(7) Check the antecedents and consequences of the concept of interest.
(8) Present empirical criteria for the concept of interest and draw conclusions for the analyzed contents.

### 2.3. Data Collection

For this study, data were collected through a literature search from 2000 to 2019, when the concept of clinical decision making was discussed in nursing based on the study by Hamm [16] on the intuition and analysis of clinical decision making. To identify the attributes, antecedents, and consequences of the concept of nurses’ clinical decision making, various domestic studies were first searched through different search engines, including the National Assembly Library and the National Digital Science Library (NDSL) provided by the Korea Institute of Science and Technology Information (KISTI), and the Research Information Sharing Service (RISS) provided by the Korea Education and Research Information Service (KERIS). The keywords that were used for the literature search included “clinical decision making,” “clinical intuition”, and “nurse decision-making ability.”

In addition, for the foreign literature, the studies published in English were searched through different search engines, including ProQuest, PubMed, MEDLINE, and CINAHL, using the keywords “clinical decision making,” “clinical intuition”, and “nurse decision-making ability.” Finally, this study was conducted based on the data collected by checking the MeSH term, title, and abstracts of the previous studies searched in the database. Studies written in English, Portuguese, and Spanish were considered for inclusion in this review.

**Figure 1:**
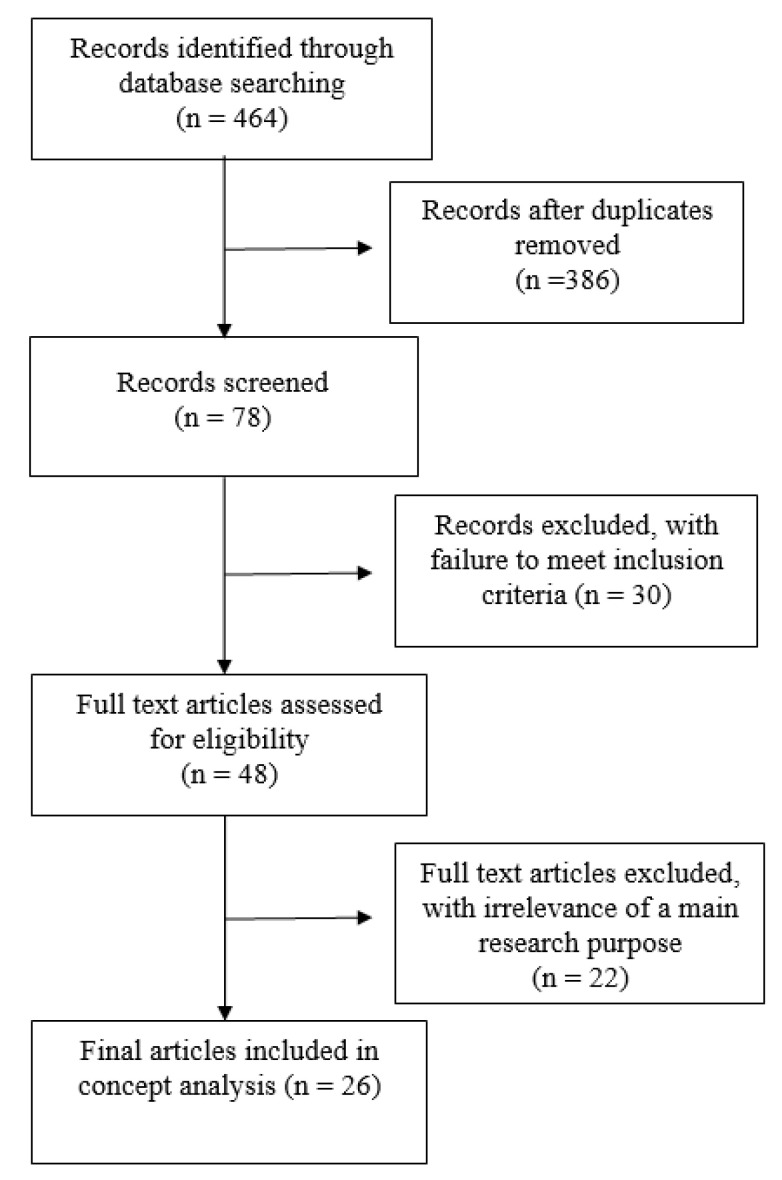
Flow diagram.

The search string was as follows: PubMed (((Nursing [MeSH Terms]) OR (nurse [Title/Abstract])) AND (((Clinical Decision Making [MeSH Terms]) OR (decision making [Title/Abstract])) OR (clinical intuition [Title/Abstract])) OR (decision making ability [Title/Abstract]))) AND ((concept [Title/Abstract]) OR (theory [Title/Abstract])) The filters were: Custom Range from 2000 to 2019, English, Portuguese, Spanish, MEDLINE (Figure 1).

### 2.4. Literature Analysis

Inclusion criteria of the literature search included the following:

- Articles from 2000 to 2019 in which the concept of nurses’ clinical decision making was discussed;
- Among the articles written in Korean and English, those whose full text had been confirmed;
- Studies conducted by investigating the concept of clinical decision making with its correlations with general characteristics and various variables; and
- Articles finally published in academic journals after being peer-reviewed.

In the previous literature, the data were repeatedly analyzed to clarify the concept of nurses’ clinical decision making. The overall concept and characteristics of nurses’ clinical decision making were identified, the factors (attributes, antecedents, and consequences) corresponding to the conceptual analysis method of Walker and Avant [11] were listed, and the related data were clearly classified and organized. Walker and Avant [11] stated that in the process of analyzing the data, the concepts appearing in the literature are to be matched, and the contents appropriate for them should be directly checked for exclusion if there are any final inconsistencies. In addition, these researchers emphasized the important insights about the concept of interest and mentioned that appropriate cases are needed to explain the concept of interest.

### 2.5. Ethical Considerations

This study was approved by the Institutional Review Board in D University.

## 3. Results

### 3.1. Dictionary Definition of the Concept

Clinical decision making requires the intuition necessary to prevent disease and promote the patient’s health under certain principles. It is the ability to cope with and resolve the patient’s behavior, which means the power to handle the nursing task [6,17].

Clinical decision making is a cognitive process in which nurses identify problems that directly or indirectly affect patients, find, and select appropriate alternatives [18]. This is a core competency that allows judgments to be made using various forms of patient information and is the basis for clinical practice [19].

### 3.2. Attributes of Korean Nurses’ Clinical Decision Making

Walker and Avant [11] stated that clarifying the attributes of the concept of interest is an important process for clearly analyzing the concept and is essential for understanding the characteristics of the concept of interest. In this study, the conceptual analysis method of Walker and Avant [11] was used to come up with a new concept or term for the clinical decision making of nurses. The interrelationships and structures of the attributes of the concepts of interest identified in previous literature were reorganized. The conceptual definitions presented in the study are concepts that contain major attributes related to the clinical decision-making ability of nurses that have already been published in journals. They were reviewed and prepared by three experts with experience in the analysis of nursing concepts, including the author of this study. For the definitions that include implicit meaning in the concept analysis process, there may be differences in interpretation depending on the viewpoint of the researcher. In addition, the concept analysis involved collecting data on the definition part that the researcher considered to be representative among the concepts contained in the journal according to research requirements and selecting the final sentence via consultation within a group of researchers with knowledge and experience in the field. Therefore, the author tried to clarify and reconstruct the main attributes from the most objective viewpoint in the analysis process of the concept of clinical decision-making ability shown in the previous literature. In this study, the process of classifying the attributes of nurses’ clinical decision making is illustrated as follows (Table 1).

#### 3.2.1. Clinical Reasoning

Clinical reasoning, the first attribute of nurses’ clinical decision making, means holistic and comprehensive thinking that can solve the diverse and complex health problems of patients. It refers to the problem-solving ability needed to recognize the patient’s health problems and predict the risks or benefits of the patient based on clinical intuition and an analytical approach [20,21,22]. This is a holistic thinking ability essential for nurses that leads to the result of professional assessment and resetting, thereby having a positive effect on the patient’s recovery and being exerted as a form of being connected with each other [3,22,23].

#### 3.2.2. Choosing and Applying Challenging Alternatives

Choosing and applying challenging alternatives, the second attribute of nurses’ clinical decision making, can be said to be a nurse’s resource that enables him or her to have confidence in the patient’s health problem despite the changes it is undergoing, and to seek an alternative to achieve desirable results [1,24]. In most studies, the nurses’ selection and application of challenging alternatives based on their clinical experience corrected the patients’ health problems, and the nurses themselves induced favorable adaptations to clinical practice with open and positive values [1,4,25,26].

#### 3.2.3. Professional Assessment and Resetting

Professional assessment and resetting, the third attribute of nurses’ clinical decision making, are powerful activities that can allow nurses to actively and independently identify when a patient’s complex health situation needs to be corrected or when a difficult problem or emergency situation is encountered [12,26]. It refers to the redistribution of resources by collecting data on the priority health needs of the patient and his or her family and on the patient’s disease, and revising the results [12,27]. This is an important factor that enables nurses to flexibly cope with nursing in the clinical practice field [25], and to eventually share their opinions with their fellow medical staff to form a social network. Above all, it is an essential element for improving nurses’ nursing capacity [1,25].

### 3.3. Cases of the Identified Attributes of Korean Nurses’ Clinical Decision Making

Walker and Avant [11] stated that the model case is a desirable case that includes the attributes of the concept of interest while the similar case is related to the concept of interest but with a different meaning because it does not contain the important attributes of the concept of interest. They also mentioned that the boundary case is a case that includes only some of the attributes of the concept of interest while the opposite case is a case that does not include any of the attributes of the concept of interest.

The attributes of nurses’ clinical decision making through this study were found to be clinical reasoning, choosing and applying challenging alternatives, and professional assessment and resetting.

#### 3.3.1. Model Case

Oh, a fourth-year nurse in the emergency department, learned from the 119 Situation Room that a male patient in his 40s was being transferred to the hospital due to a fall at a construction site. The patient fell on the floor after losing his footing while working at the apartment construction site. Fortunately, he was conscious, but his vital signs were unstable. Since arrival, the patient (Kim) experienced a decrease in consciousness and loss of sensation in both legs. Among the patient’s vital signs, blood pressure was unstable at 80/50 mmHg and hemoglobin level was 10.0 g/dL. The nurse suspected that the patient had brain and intraperitoneal hemorrhage and cervical spine injury as the patient fell to the floor without wearing protective equipment during work. Nurse Oh was concerned about general paralysis due to the cervical spine injury and deterioration of consciousness caused by the massive bleeding in the abdominal cavity. Sufficient fluid and blood were supplied to the patient as an initial treatment and careful monitoring of the patient’s response was conducted while periodically checking for sensory and motor responses. In addition, Nurse Oh included the administration of narcotic analgesics and surgical preparation in the nursing plan for the likelihood of emergency surgery and reduction of secondary pain from severe pain. For rapid treatment, the department related to trauma, which may have occurred to the patient, was called for consultation, while treatment plans, such as blood test and imaging test results, were shared. After receiving a call from the hospital’s trauma team and conducting related consultations, a cervical spine number two injury and hemoperitoneum due to fall were diagnosed, and emergency neurosurgery and trauma surgery were decided upon. As a member of the treatment team, Nurse Oh shared the information on the ongoing examination, treatment status, and condition of the patient with the treatment team. Through prompt preparation for surgery and transfer of the patient to the anesthesia department, the patient safely underwent surgery and was moved to the intensive care unit.

In this case, the nurse predicted the patient’s unstable state through clinical intuition and analytical power and was trying to solve the patient’s health problem by carefully observing the subject’s reaction to find a more active alternative. In particular, it can be seen that nurses are flexible and actively coping with performing nursing for patients through professional assessment and interaction with fellow medical staff.

#### 3.3.2. Additional Case

##### Similar Case

Kim, a fifth-year nurse in the medical ward, was in charge of a 50-year-old female patient with suspected gastrointestinal bleeding. The patient complained of persistent nausea but had no vomiting symptoms. She had a pale face and reported pain behind her neck every time she swallowed. Nurse Kim calmly asked her colleagues for help, stabilized the patient comfortably, and monitored the patient’s vital signs and overall health.

##### Boundary Case

A 30-year-old female patient suspected of having acute appendicitis asked for a pain reliever from Park, a third-year nurse in the emergency department, because she had a stomachache and could not lie on her back. Nurse Park did not perform physical examinations such as “rebound tenderness” but asked the patient to point to the painful area because they needed to prescribe a medicine for her, while telling her to be patient.

##### Opposite Case

Lee, a fourth-year nurse in the surgical ward, took over a 20-year-old male patient from Ku, a third-year nurse, but forgot the takeover details about the patient that nurse Ku had told her when a 40-year-old female patient vomited and showed unstable blood pressure. The 20-year-old male patient, who did not have a caregiver, urgently asked nurse Lee to help him go to the restroom, but nurse Lee did not respond. The patient fell while coming down from the bed.

### 3.4. Antecedents and Consequences of Korean Nurses’ Clinical Decision Making

According to Walker and Avant [11], antecedents refer to events or phenomena that can be grasped through the concept of past events, or phenomena that occurred prior to the occurrence of the concept. They claim that antecedents help in determining the attributes of the concept of interest.

**Figure 2:**
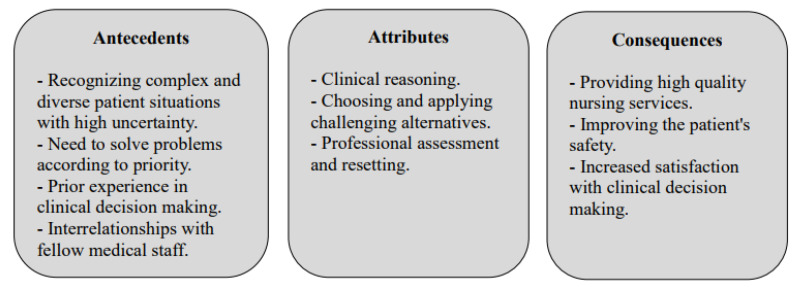
Conceptual diagram of clinical decision making.

The antecedents of nurses’ clinical decision making typically appear in the literature as the recognition of complex and diverse patient situations with high uncertainty, the need to solve problems according to priorities, prior experience in clinical decision making, and interaction with one’s fellow medical staff. The consequences of nurses’ clinical decision making, on the other hand, were found to be the provision of high-quality nursing services, improvement of the patient’s comfort, and increased patient satisfaction with the nurse’s clinical decision-making ability. The antecedents and consequences of nurses’ clinical decision making that were found in this study through literature search are as follows (Figure 2).

First, for the antecedents of nurses’ clinical decision making, clinical judgment experience regarding the patient’s health status, which is constantly changing and has high uncertainty and complexity, is important [15,20,28]. Priority is given to the patient’s health problem, and the problem is predicted and treated through the nurse’s interaction with his or her fellow medical staff [29,30]. At the same time, improperly recognizing the situation and misidentifying the priorities can have a very negative impact on the patient’s health problem [14,29,30,31].

Second, for the consequences of nurses’ clinical decision making, the patient’s psychological well-being and comfort improves when provided with nursing. Thus, nurses’ clinical decision-making ability acts as a positive predictor [32] and makes patients think that they are receiving high-quality nursing services [8,17]. As a result, nurses develop a sense of accomplishment and gain high satisfaction with their clinical decision making [15,20,33].

### 3.5. Empirical Criteria for Korean Nurses’ Clinical Decision Making

The empirical criteria are directly related to the defined characteristics or attributes of the concept of interest, thus aiding in their recognition [11].

In this study, the empirical criteria for nurses’ clinical decision making are the key competencies that nurses need to be able to professionally grasp the priority problems of patients so as to resolve their health problems and to enhance their comfort, which nurses can acquire through prior nursing knowledge and experience and by improving their interactions with their fellow medical staff.

All the previous relevant studies cited proper clinical decision making as a key competency of nurses [17,20,22,27,32]. Proper clinical decision making by nurses has helped nurses flexibly cope with the changes in the clinical practice field and in the medical environment [9,17,21], and has helped them accomplish their tasks in crisis situations. Moreover, when nurses encounter stress, adversity, or difficult problems, proper clinical decision making can give them the driving force to cause healthy behavior on the part of the patient [31,33,34]. After all, proper clinical decision making, which can be considered the key competency of nurses, can improve nurses’ self-efficacy and can give them a sense of accomplishment, in addition to increasing the satisfaction of the society with the country’s medical system, and maintains patients’ well-being, which can boost patients’ comfort and their desire for social adaptation [1,7,28,29].

## 4. Discussion

As a result of the conceptual analysis that was conducted in this study, it was found that the chief attribute of nurses’ clinical decision making is the ability to professionally assess the patient’s condition and select a holistic alternative that can effectively solve the patient’s health problems, based on clinical reasoning ability. In addition, it was confirmed that the attributes of nurses’ clinical decision making are intertwined with one another and are regularly arranged, leading to desirable consequences in terms of improving patient comfort.

Therefore, this study intended to examine the attributes, antecedents, and consequences of nurses’ clinical decision making in a nursing flow, and to find ways to beneficially apply these to nursing practices.

For the results of this study, the first attribute of nurses’ clinical decision making, clinical reasoning ability, helps nurses gradually develop professionalism and leadership because skilled nurses have an excellent ability to interpret the patient’s problem based on their prior experience, and can cooperate seamlessly with their fellow medical staff when faced with a situation involving a patient’s highly uncertain health status [5]. Additionally, Kim and Jung [6] mentioned in their study that nurses must evenly distribute essential medical resources to be able to make the right decisions for the professional nursing of patients and must check the patient information based on clinical reasoning developed from past work performance [4]. Therefore, it is considered that nurses need the ability to utilize consistent and predictable resources for patients by modifying and confirming the process of systematic and collective clinical reasoning in establishing a series of nursing plans for patients.

Kim et al. [4] stated that nursing with a challenging sense of purpose and with the desire and capability of finding the right nursing alternative for the patient could improve nurses’ unique work performance, and above all, maximize their efficiency in carrying out their nursing tasks. This supports the consequence of the second attribute of nurses’ clinical decision making: choosing and applying challenging alternatives. The studies of Jeffery et al. [24] and of Mun and Kim [1] explained that challenging nursing skills could be gradually developed more efficiently and proficiently as the idea that nursing is a profession is enhanced, and as nurses’ interaction with their fellow medical staff is improved while performing nursing [1,25]. Therefore, nurses’ healthy exchange of opinions with their fellow medical staff can help them successfully perform their nursing tasks and can help increase their professionalism and job satisfaction.

Proper clinical decision making is driven by nurses’ nursing knowledge and prudence acquired from their previous experience. Mun and Kim [1] and Oh and Kim [7] mentioned that nurses can make good judgments when assessing a patient professionally, based on their discretion and values. This supports the third attribute of nurses’ clinical decision making: professional assessment and resetting. In the studies of Kim et al. [4] and Mun and Kim [1], nurses’ careful and professional assessment allowed them to continuously modify the nursing plan that they had made within the unique work process of nursing, thereby enabling them to effectively address their patients’ health problems while increasing their sense of accomplishment as nurses [20,21,25,27].

However, despite the definition of the concept verified in various literature on the decision-making ability of these nurses, Korean nurses are still focusing on basic bedside care because of the legal feature that care should be performed under the guidance and supervision of doctors with a common goal in mutual relations with doctors [4,7]. This is seen as a clear difference from nurses of other countries in that Korean nurses do not have the right to choose all the actions they perform, which significantly decreases the autonomy of nurses as experts and acts as a factor that lowers their morale [3,4,7,8].

Based on the results of this study, among the attributes of Korean nurses’ clinical decision making that were identified, clinical reasoning, choosing and applying challenging alternatives, and professional assessment and resetting can increase nurses’ self-esteem and satisfaction with their clinical decision-making ability, and can make them actively participate in the nursing work environment. This acts as a leading factor that positively affects the correct coping ability for nurses in an urgent situation of caring for a patient’s constantly changing health condition [29,30,31]. In addition, nurses’ clinical judgment ability finally shines as a result of maximizing the patient’s comfort through therapeutic communication with fellow medical staff [32].

Ultimately, nurses’ clinical decision-making ability can increase insight into the patient’s condition, and as a result, a sense of accomplishment can be seen. Above all, the clinical decision-making ability of nurses suggests specific ways to achieve nursing performance and informs them that they can grow into talented nurses who can demonstrate their competence.

The autonomous and active efforts of nurses to improve their nursing work environment will enable them to derive the key competencies that are essential for nurses even in a changing health crisis situation. They will lead to an improvement in the quality of nursing services delivered and can also contribute to the overall enhancement of patient comfort and to an exploration of health policy.

In the future, based on the results of this study, research on clinical decision making considering the characteristics of the nursing profession, identification of additional variables affecting Korean nurses’ clinical decision making, and development of nursing tools for clinical decision making are suggested. In addition, it is necessary to develop a nursing intervention program for Korean nurses’ clinical decision making, and to conduct experimental studies to verify its effectiveness.

A limitation of this concept analysis is that the data sources used for the analysis were limited to the disciplines of nursing in clinical practice. Reviewing the literature of other disciplines may illustrate similarities and differences in the use of the concept across a broad field.

## 5. Conclusions

A nurse’s clinical decision making is the nurse’s intuitive ability to facilitate a healthy life for the patient, and it is to actively break through the crisis situation that appears to the patient and make a desirable choice for a challenging alternative. In addition, the nurse professionally assesses and solves problems for the safety of the patient.

The conceptual attributes of nurses’ clinical decision making through the results of this study had slightly different characteristics, but they were organically related to each other.

This study is significant in that the unclear conceptual difference of Korean nurses’ clinical decision making in all fields of nursing phenomena, as found through a literature search, was resolved, allowing for a clear understanding of the multidimensional attributes of clinical decision making. In addition, the concept of Korean nurses’ clinical decision making provided basic data for the improvement of nursing practices and patient comfort, and for further nursing research and health policy.

## Figures and Tables

**Table 1:** Attributes of clinical decision making.

Author (Year)	Concept Description	Attributes
Hwang (2018)	Assumption of the disease through the collected information and prediction of the treatment and outcome	(1)
Son and Kim (2018)	Solving of problems based on nurses’ experience and knowledge	(1)
Hawkins, Elder, and Paul (2013)	Integrated analysis and interpretation of nurses’ clinical practice knowledge and of the information collected from the patient to select an alternative	(1)
Mun and Sim (2018)	Critical thinking for actual clinical situations	(1)
Wang, Chien, and Twinn (2012)	Selection of alternatives through reasonable judgment from limited information	(2)
Park (2018)	Reasonable judgment to identify the patient’s problem and select an alternative suitable for nursing performance	(2)
Kim, Cho, and Kim (2015)	Selection of appropriate alternatives related to the discovered health problems	(2), (3)
Mun and Kim (2016)	Detection of the characteristic symptoms of the expected disease through a physical examination, and selection and application of an alternative nursing intervention that suits the priority	(2), (3)
Forbes, Surdeneau, Jansen, and Carrington (2013)	Search for and application of solutions to individual problems through fast and accurate analysis	(2), (3)
Jeffery, Novak, Kennedy, Dietrich, and Mion (2017)	Making of appropriate plans and systematic tackling of complex tasks	(2), (3)
Chung and Song (2011)	Assessment and redistribution of resources based on disease-related information	(3)
Lee, Gang, and Jung (2013)	Scientific thinking and rational decision making by understanding the situation with nurses’ clinical knowledge and collected information	(3)
Lim (2016)	Deciding on an action based on nurses’ patient assessment results according to the priority problems	(3)
Mun (2012)	Confirmation of the disease through reasoning based on physical examination and objective data	(3)

(1) Clinical reasoning, (2) Choosing and applying challenging alternatives, (3) Professional assessment and resetting.

## Data Availability

No new data were created or analyzed in this study. Data sharing is not applicable to this article.
